# Smokeless Tobacco and Public Health in Bangladesh

**DOI:** 10.4103/ijph.IJPH_233_17

**Published:** 2017-09

**Authors:** Rumana Huque, M. Mostafa Zaman, Syed Mahfuzul Huq, Dhirendra N. Sinha

**Affiliations:** 1 Professor, Department of Economics, University of Dhaka, Dhaka, Bangladesh; 2 Adviser, Research and Publication Unit, World Health Organization, Dhaka, Bangladesh; 3 National Professional Officer (Tobacco Control), World Health Organization, Dhaka, Bangladesh; 4 Director, Department of Tobacco Control, School of Preventive Oncology, Patna, Bihar, India

**Keywords:** Bangladesh, determinants of smokeless tobacco use, prevalence of smokeless tobacco, smokeless tobacco, tobacco tax

## Abstract

Despite the high prevalence of smokeless tobacco (SLT) use among adults in Bangladesh, SLT was not included in the Tobacco Control Law till 2013. Information on SLT use among Bangladeshi people is inadequate for policymaking and implementing effective control measures. With the aim to identify the prevalence and trends of different SLT products, health and economic impacts, manufacture, and sale of and policies related to SLT in Bangladesh, we carried out a literature review, which involved literature search, data extraction, and synthesis. Evidence suggests that in Bangladesh, SLTs range from unprocessed to processed or manufactured products including *Sada Pata, Zarda, Gul*, and *Khoinee*. Over 27% of Bangladeshi adults aged 15 years and older use SLT in one form or other. SLT use is associated with age, sex, education, and socioeconomic status. SLT consumption has reportedly been associated with increased prevalence of heart diseases, stroke, and oral cancer and led to around 320,000 disability adjusted life years lost in Bangladesh in 2010. No cessation service is available for SLT users in public facilities. Compared to cigarettes, taxation on SLT remains low in Bangladesh. The amendment made in Tobacco Control Law in 2013 requires graphic health warnings to cover 50% of SLT packaging, ban on advertisement of SLT products, and restriction to sale to minors. However, implementation of the law is weak. As the use of SLT is culturally accepted in Bangladesh, culturally appropriate public awareness program is required to curb SLT use along with increased tax and cessation services.

## Introduction

The term “smokeless tobacco” (SLT) implies the use of any unburned tobacco in the finished product[1] that can be consumed orally or nasally. There is a great diversity of SLT products and their use patterns across the globe, and it refers to more than thirty different products, broadly categorized as “spit tobacco” or “chewing tobacco.”[2,3] Its use is relatively high in South and South-East Asia where one-third of tobacco is consumed in smokeless form.[4] Tobacco is being chewed in multiple forms and modes in South Asia such as betel leaf with areca nut, betel leaf alone, *Zarda* and *Gul*,[5] and the pattern of consumption varies across countries by differing sociocultural norms, habits, peer influences, availability, accessibility, and legislations in place.

Current SLT use prevalence is especially high (>15%) among adults in Myanmar, Bangladesh, India, Bhutan, Nepal, Pakistan, and Sri Lanka.[6] A quarter of adults in Bangladesh and India use SLT.[7] SLT use is considered as the predominant form of tobacco use in Bangladesh, whereas 23% of adults smoke and 27% of adults use SLT.[6] Despite its popularity, there is little information available on SLT, making it difficult for policymakers to develop and implement effective control measures for these products. Against this backdrop, we carried out a review to explore the situation of SLT manufacture, trade, tax structure, and consumption in Bangladesh. More specifically, we explored various types of SLT products commonly available, the patterns of consumption, the prevalence of its use among populations, health and economic impacts, the key determinants and drivers that are influencing its use, and the legislation, policies, and tax structure related to SLT in Bangladesh.

## Methods

We carried out a literature review to identify the prevalence and trends, health and economic impacts, manufacture, distribution and sale of SLT, and control measures in Bangladesh. A range of published written materials including books, journal articles, and research reports was reviewed. The review process involved three distinct stages: literature search, data extraction, and synthesis. A series of key search terms was used in the review process, which were broadly grouped into the following themes: “SLT,” “chewing tobacco,” “tobacco,” “SLT in Bangladesh,” and “SLT in South Asia.” PubMed and ResearchGate were the major databases searched for this purpose. Websites of WHO, Government of Bangladesh, and relevant research organizations had been searched. Reference lists of published articles/report/books also helped to identify relevant articles and documents. Filtering abstracts or executive summaries of all materials identified were reviewed. All abstracts were assessed against predefined inclusion and exclusion criteria. The search strategy was guided by the understanding of languages (English only because literature in Bengali is unheard of) and the period (1990–2016) restrictions.

## Results

### Types and prevalence of smokeless tobacco use in Bangladesh

A diverse range of SLT products is used in Bangladesh including unprocessed, processed, or manufactured products. They vary in their composition, methods of preparation, and consumption pattern [Table 1].[7]

The prevalence of tobacco use was found 43.3% among the adult population (aged 15 and above), of which SLT use was higher at 27.2% [Table 2], exceeding the prevalence of smoking (23%) in Bangladesh.[8]

Other studies also suggest that dry tobacco leaf (*Sada Pata*), *Zarda*, and *Gul* were found being popularly used in country[13,14] and in terms of quantity of sale in Bangladesh.[15] Different brands of *Zarda* and *Gul* are available in local markets, of which six brands of *Zarda* and three brands of *Gul* are popular.[16]

Although the prevalence of smoking is high among males (26.4%) than females (1.5%), use of SLT is slightly higher among females than among males in Bangladesh.[8,17–19] SLT use is high among both men (44%) and women (42.5%) aged 55–64 years.[20] The average age at onset of SLT consumption was found 31.5 years.[21] The prevalence of current use of SLT among boys is 7.1% and among girls is 3.7% aged 13–15 years. A recent study suggests a significantly increasing trend in the prevalence of current SLT use among Bangladeshi men aged 15–49 years (20.2%–23%, *P* = 0.03).[22]

The average length of SLT use varies by types. Average daily use of *Sada Pata* (7.1 times/day) was found higher than that of *Zarda* (6.3) and *Gul* (4.5).[11] Daily betel quid (*Paan*) chewing frequency was higher among those who chewed with tobacco (mean 5.6, standard deviation 3.9) than those who chewed without tobacco.[21]

SLTs in Bangladesh are also used in combination with smoking products. Dual users constituted 20% among all tobacco users.[13,20] Prevalence of “smoking only,” “smokeless only,” and “dual use” of tobacco was found 40.6%, 15.2%, and 14.2%, respectively.[13] The average age of dual users was found 46.7 years old compared to 43.4 and 52.3 years for smokers and SLT users, respectively.[13]

### Price of smokeless tobacco in Bangladesh

Price of the SLT products varies. The cheaper variety of *Zarda* is sold for <0.60 Taka per gram, which is comparable to the *biri* price per stick, and the higher-price variety sells for 0.60–1.00 Taka per gram, which is higher than *biri* price per stick.[23] On average, the price of *Zarda* per gram is less than half of the price per stick of the cheapest brand of cigarette. The price of *Gul* is relatively skewed centering around 0.10 Taka per gram.[23]

### Socioeconomic determinants of smokeless tobacco use

The use of SLT was found to be associated with age, sex, education, and socioeconomic status.[8,14,17,20,21,24] The SLT use rate increases persistently with age, ranging from 6.6% in the age group of 15–24 years to 56.4% in the age group of 65 years and above.[8]

Over half of SLT users in Bangladesh have no formal education. Individuals belonging to households with educated heads or owning more than 200 decimals of land consumed less tobacco in proportion to those with illiterate heads or were landless.[20] Farmers (33.8%), the unemployed (31.9%), and laborers (30.9%) use SLT at a higher rate.[21]

Dual users also had lower educational achievement, rural residence, lower intake of fruit, and higher intake of alcohol.[13,18] They were socioeconomically deprived as measured by wealth quartiles constructed out of household assets and more undernourished as indicated by a thin body mass index compared to nonusers and smokers.[8,13,18]

Global Adult Tobacco Survey (2009) suggests that SLT use is more prevalent in rural (28.8%) than urban areas (22.5%),[8] whereas Flora *et al.* observed that SLT was 1.5 times more likely to occur in rural residents and *Gul* usage was 3.6 times more likely to occur in urban residents.[25]

The quantity demanded of SLT products depends not only on price but also on other factors such as seasonality, the time of month, and festivals. During winter, SLT sales increase whereas it declines during the summer.[16]

### Smokeless tobacco-related mortality and morbidity

Although a large number of studies assessed the adverse health effects associated with smoking tobacco, only a few researches have been focused on SLT-related mortality and morbidity in Bangladesh. SLT consumption has been found to be associated with increased prevalence of high blood pressure in the adult male in rural Bangladesh.[21,26] Chewing betel quid has been linked to obesity and cardiovascular disease,[21] oral cancer and stroke,[5,18] and hypertension.[21]

Dual use of tobacco, especially in men, increases the risk of some cancers and carries a higher risk of ischemic heart disease (IHD) in Bangladeshi and other populations.[13] A population-based study in Bangladesh has shown that tobacco use and hypertension are significant (*P* < 0.05) factors for IHD in rural and agricultural free living population with traditional lifestyle and thin body mass index.[27] Zaman *et al.* suggest that the overall related risks and population attributable risks of diseases vary by gender for SLT usage [Table 3].[9]

In 2010, SLT use led to a total of 320,000 disability-adjusted life years lost and 13,329 deaths due to cancers of mouth, pharynx, and esophagus and ischemic heart disease in Bangladesh.[7]

### Availability of cessation services

Despite high prevalence of SLT use and associated morbidity and mortality, there are no cessation services available for SLT users in different tiers at public health-care facilities in Bangladesh. Huque *et al.* showed that cotinine concentration among SLT users in Bangladesh is very high, thus pointing toward a high level of addiction and importance of effective tobacco control policies to help SLT cessation.[28]

A recent study showed that tobacco cessation by a simple counseling can be very successful in a village level clinic, especially for SLT use, where its prevalence dropped from 33.2% to 0.4% from 1^st^ to 5^th^ counseling session.[29] This has a high potential generalization that community clinics in Bangladesh would be important organizations for tobacco cessation services. National Heart Foundation, a leading hospital and research institute in Bangladesh, jointly with hypertension clinic has started a tobacco cessation clinic. Its experience is yet to be reported.

### Attitudes and misbelieves

In Bangladesh and other South Asian countries, though traditional values and social norms do not favor smoking by the young or by women, there is no such taboo against SLT,[16] being incorporated in traditional values, spirituality, beliefs, festivals, lifestyle, and rituals such as marriage and popular entertainment.[2,30] An estimated 20%–30% of women in the rural areas use SLT as a tradition,[31] serve it to their guests in cultural celebrations,[30] and equate it to confectionary.[2]

Many myths and misconceptions are attached to SLT use which are deeply rooted, especially in the rural populations such as SLT helps to aid digestion if taken after meal, pain relief, curing toothache, headache and stomach ache, to cope with boredom, frustration and for mental relaxation purposes, relieving tension, aiding concentration, combating bad breath, protection from snake and scorpion venom, and its use is less harmful than smoking.[32–35] Some forms are believed to make women feel better from morning sickness during pregnancy. Curiosity, peer pressure, and offers by friends and acquaintances contribute to initiation of SLT use.[14] Some parents even encourage their children to use SLT.[2] This is reflected in Global Youth Tobacco Survey, indicating a high prevalence of SLT use among 13–15 year olds in Bangladesh.[19]

### Supply chain of smokeless tobacco

A number of actors are involved in the production and sale of processed SLT products including farmers, raw tobacco retailers, manufacturers, wholesaler/wholesaler dealers, and SLT retailers. However, the supply chain is not straight forward as many of the actors are interlinked.[15]

### Production, export, import, and illicit trade

A significant proportion of smuggled and counterfeit SLT products are available in the market in Bangladesh. Siddiqi *et al.* (2015) suggest that 88% of the SLT products sold by vendors surveyed in a study were produced locally whereas the rest were products from neighboring countries.[15] Tax and custom evasion led to rapid increase in smuggling of SLT products across India and Nepal border, after the introduction of value added tax (VAT) in 1997.[36] One study indicates that about 14% of the SLT products sold in the country are foreign smuggled, 45% of which are smuggled through land ports whereas 26% are smuggled through air ports.[37] Increased availability of illicit products lowers consumer prices through tax evasion, which in turn increases consumption and threatens both tobacco control efforts and excise tax collection by governments.[38]

### Regulation and legislation

Bangladesh has constantly shown commitment toward tobacco control. Tobacco Control Law was enacted in 2005 and was further strengthened in 2013 by including SLT and various other provisions.[39] In real sense, the comprehensive movement against SLT started only in 2013 because SLT was not covered by the 2005 version of the act.[39] The impacts of the 2013 amended version of act are yet to be visible because its rules have been approved only in March 2015.

Various legislations exist in Bangladesh, which can indirectly affect SLT tobacco production and consumption. For example, Metropolitan Police Ordinances which prohibit spitting at public places can be used to curb SLT use as SLT users frequently spit. However, the enforcement of the law is currently weak.

A recent study shows that though 53% of SLT products collected from the markets had a written health warning, 44% among them had low visibility due to very small font, 22% had warning in English only, 11% had these tactfully hidden in the packaging, and 11% had misleading information.[15] Nearly 11.8% of products had a label saying “not suitable for children,” whereas 41.2% of products printed ingredients on their labels, of which only 57% mentioned “tobacco” as an ingredient.[15]

Inadequate knowledge about existing law relating to SLT, inadequate training of law enforcers, unclear roles and responsibilities of different government departments, and inadequate resources for enforcement are some of the barriers in implementing existing legislations.[39] In Bangladesh, Standard and Testing Institute for food safety is not responsible for checking and monitoring SLT in food products, which needs attention of the policymakers and respective authorities.[39]

### Media image

SLT advertising is ban in Bangladesh. The current law prohibits advertising tobacco products including SLT in print (local and international magazines and newspapers), and electronic media (national and international radio and television and internet), and in any other forms such as advertising at point of sale and using billboards and outdoor advertising. Current law requires fines for violations of direct advertising bans.

### Tax

One of the major challenges in regulating SLT products is their low tax and the consequent low price, thus making it readily accessible, especially to the youth. Compared to cigarettes, taxation on SLT remained generally low in Bangladesh [Table 4]. However, the government recently increased excise tax on SLT. Currently, tax on cigarette ranges from 66% to 80% of the retail price, whereas tax on *Zarda* and *Gul* is 116%, thereby the tax rate on SLT looks good. However, the flaw is that the tax base for SLT is “ex-factory price” – which is far less than the retail price. This makes tax burden on SLT real low. National Board of Revenue collects tax from twenty SLT manufacturers only,[16] whereas many SLT manufacturers are not registered and doing business illegally, and they remain out of paying taxes and VAT to the government.

The complex ad valorem tax structure and weak tax administration result in high tax evasion. Sale of SLT products in informal establishments, often in unpacked forms, and diverse nature of the manufactured products in different-sized pack also increase the possibility of tax evasion. Absence of annual systematic inflation-adjusted increase in tobacco taxation, inadequate understanding of tax policies within health ministry, poor coordination between governmental departments, lack of appropriate training and resources within implementing agencies, lack of knowledge with respect to the absolute and relative prices of tobacco products (smoked and smokeless), and lack of recognition of the implications of the current price structure of tobacco products for overall tobacco consumption are some of the other constraints identified in this policy area.[23]

Nargis *et al.*, 2014, estimated the price elasticity of lower price brands of *Zarda* at ‒ 0.64 and of higher priced brands at − 0.39, and the cross price elasticity of *Zarda* with respect to cigarette price at 0.35. This implies that the tax increase on SLT needs to be greater than the tax increase on SLT to bridge the wide price differential between the two types of products that currently encourages downward substitution from smoked to SLT and discourages quitting behavior.[23]

## Discussion and Way Forward

Tobacco use is intimately linked to poverty either as a cause or effect. Therefore, SLT control strategies should be an integral part of poverty reduction to establish equity and social justice. Despite high prevalence of SLT use, little attention has been paid by policymakers and researchers on the issue in Bangladesh. We carried out a rapid assessment of the SLT use in Bangladesh through document review.

### Prevention of smokeless tobacco consumption through gender-based culturally accepted strategies

Although evidence is limited in Bangladesh, a number of studies showed strong and consistent evidence to indicate significant risk of oral cancer and pharyngeal cancer, oral neoplasia, esophageal cancer, and pancreatic cancer, poor oro-dental health, risk of hypertension and cardiovascular diseases, and adverse effects on reproductive health (especially during pregnancy with birth complications, fetal loss, low birth weight, and prematurity) with SLT use in India and other Asian countries.[2,40–42] The risk of these conditions is found to increase with increasing dosage and frequency of SLT use. Evidence also suggests that SLT caused 7.1% of deaths from all medical causes in India.[43]

Prevention of SLT consumption could, therefore, be an important intervention in preventing the ongoing upswing in the prevalence of these diseases.

Several studies confirmed that SLT use is high among women in Bangladesh which is similar in India, Thailand, Cambodia, Malaysia, Vietnam, and some African countries, such as South Africa, Mauritania, and Sierra Leone.[6,24] The inverse relationship between socioeconomic status and tobacco consumption found in Bangladesh is also consistent with findings in other South Asian countries. This highlights the importance of developing different tobacco control strategies to target men and women and for different socioeconomic status. As the use of SLT is culturally accepted in Bangladesh, culturally appropriate public awareness is required to curb SLT.[18]

### Impose high tax on smokeless tobacco

Easy availability, low price and affordability, misconceptions regarding its useful health effects, increasing population, lack of tobacco control regulations, and weak enforcement of existing regulations contribute to the increase in tobacco consumption in Bangladesh.

Bangladesh would require effective tobacco control programs to combat the tobacco epidemic along with national plans of actions. Imposition of higher tax on SLT and changing the tax base for SLT to retail price is essential. Measures to bring all the tobacco products under tax and price measures should be sought for, to avoid substitution of one tobacco product by another. Moreover, a specific excise system replacing the existing ad valorem excise tax can substantially contribute to the revenue collection performance from SLT products.[23] Creation of public awareness about health hazards of SLT tobacco is also necessary.

### Mapping and developing surveillance system

Availability of locally reliable population-based data on tobacco use burden and tobacco-attributable morbidity and mortality, as well as their predictors, are helpful in advocating for national tobacco control strategies. Understanding the population burden of tobacco use contributes to the development of effective interventions and policies to reduce tobacco use; measuring changes in population. Such data can help in determining specific strategies for intervention.[18]

A mapping of SLT manufacturers is required to understand the market share of SLT and the coverage for tax. The mapping should be done by administrative area and by type and volume of SLT production.

### Research need

It is important to identify the existing subsidies or incentives for tobacco cultivation in Bangladesh and the factors that influence increased land use for cultivation of tobacco. Government should take initiative to encourage farmers in producing other crops. Estimating the illicit trade of SLT products and the potential ways to stop illicit trade needs to be investigated.

Lack of monitoring and surveillance data in relation to SLT use and its impacts and unknown chemical contents of different types of SLT products available in Bangladesh are constraining designing of appropriate strategy and intervention for reducing SLT consumption. A comprehensive analysis of the contents of locally produced different types of SLT products and harmful effects of consumption of such products is needed to monitor and regulate the emission and ingredients among all smoked and SLT products used in the country. The third International Conference on Smokeless Tobacco held in Stockholm in 2002 emphasized on research needs in assessing the chemistry and constituents of SLT products.[6] Similar analysis in India suggests significant variation in nicotine content across products[44,45] where nicotine content in SLT products ranged from 0.8 to 50.0 mg/g.[45]

There is a need for research on effectiveness and cost-effectiveness of innovative cessation treatments for SLT users in Bangladesh.

## Conclusion

Tobacco use is intimately linked to poverty either as a cause or effect. Therefore, SLT control strategies should be an integral part of poverty reduction to establish equity and social justice. As use of SLT is culturally accepted in Bangladesh, culturally appropriate public awareness is required to curb SLT.[18]

## Figures and Tables

**Table 1:** Popular smokeless tobacco products in Bangladesh: Ingredients, forms, and consumption

SLT products[8]	Ingredients[7]	Form[7]	Consumption[7]	Prevalence (%) of SLT use among adults aged ≥15 years[8]
*Zarda*	Tobacco, lime, spices, vegetable dye, areca nut	Manufactured commercially	Chewed with betel leaf, lime, and areca nut	24.3
*Gul*	Tobacco powder, molasses	Manufactured commercially	Applied to teeth and gum	5.3
*Sada Pata*	Sun-dried or cured raw tobacco leaf	Processed but unpacked	Chewed with betel leaf, lime, and areca nut	1.8
*Khoinee*	Tobacco, slaked lime, menthol, flavorings, and areca nut	Manufactured commercially or prepared by user themselves	Kept in mouth between lips and gum	1.5
Any form of SLT		Manufactured commercially, or processed but unpacked, or prepared by user	As above	27.2

Adapted from Siddiqi *et al*., 2015; WHO, 2009. SLT: Smokeless tobacco

**Table 2:** Prevalence (%) of smokeless tobacco usage estimated in different national level studies

Indicator	Health cost study 2004 (15 years or older)[9]	STEPS 2006 (25-64 years)[10]	GATS 2009 (15 years or older)[8]	STEPS 2010 (25 years or older)[11]	STEPS 2013 (25 years or older)[12]
Percentage of men who use SLT	14.8	24.1	26.4	29.4	28.5
Percentage of women who use SLT	24.4	34.2	27.9	33.6	29.5
Percentage of men and women who use SLT	19.7	28.6	27.2	31.7	28.7

SLT: Smokeless tobacco

**Table 3:** Related risk and population attributable risk of diseases for smokeless tobacco usage from the hospital survey

Diseases	Men		Women	
	Related risks	Population attributable risk (%)	Related risks	Population attributable risk (%)
Ischemic heart disease	2.6	32.5	1.7	22.6
Stroke	2.1	23.8	2.6	41.2
Buerger’s disease	1.8	16.1	Negligible	Negligible
Oral cancer	4.9	48.1	4.7	59.3
Lung cancer	1.2	3.9	12.6	82.7
Laryngeal cancer	1.5	10.2	7.8	73.1
Chronic obstructive pulmonary disease	1.7	15.0	1.8	24.6
Pulmonary tuberculosis	1.9	18.8	3.5	51.0
All	1.5	12.9	2.0	33.1

Source: WHO, 2009

**Table 4:** Tax on smokeless tobacco in Bangladesh

Year	Tax on SLT*			Total (%)	Total tax collection from VAT and SD (million Taka)
	SD (%)	VAT (%)	Health development surcharge (%)		
2010-2011	20	15	-	35	57.2
2011-2012	30	15	-	45	79.5
2012-2013	30	15	-	45	87.5
2013-2014	30	15	-	45	106.3
2014-2015	60	15	1	76	175.5
2015-2016	60	15	1	76	69.2 (up to October, 2015)
2016-2017	100	15	1	116	

* The tax base is ex-factory price. SD: Supplementary duty, SLT: Smokeless tobacco, VAT: Value added tax
